# A Hierarchical Poisson Log-Normal Model for Network Inference from RNA Sequencing Data

**DOI:** 10.1371/journal.pone.0077503

**Published:** 2013-10-17

**Authors:** Mélina Gallopin, Andrea Rau, Florence Jaffrézic

**Affiliations:** 1 Département de Génétique Animale, INRA, Jouy-en-Josas, France; 2 Département de Génétique Animale, AgroParis Tech, Paris, France; 3 Département de Mathématiques, Université Paris-Sud 11, Orsay, France; The University of Chicago, United States of America

## Abstract

Gene network inference from transcriptomic data is an important methodological challenge and a key aspect of systems biology. Although several methods have been proposed to infer networks from microarray data, there is a need for inference methods able to model RNA-seq data, which are count-based and highly variable. In this work we propose a hierarchical Poisson log-normal model with a Lasso penalty to infer gene networks from RNA-seq data; this model has the advantage of directly modelling discrete data and accounting for inter-sample variance larger than the sample mean. Using real microRNA-seq data from breast cancer tumors and simulations, we compare this method to a regularized Gaussian graphical model on log-transformed data, and a Poisson log-linear graphical model with a Lasso penalty on power-transformed data. For data simulated with large inter-sample dispersion, the proposed model performs better than the other methods in terms of sensitivity, specificity and area under the ROC curve. These results show the necessity of methods specifically designed for gene network inference from RNA-seq data.

## Introduction

In recent years, high-throughput sequencing technology has become an essential tool for genomic studies. In particular, it allows the transcriptome to be directly sequenced (RNA sequencing), which provides count-based measures of gene expression. Typically, the first biological question arising from these data is to identify genes differently expressed across biological conditions. Because RNA-seq data are known to exhibit a large amount of variability among biological replicates, most methods for differential analysis are based either on overdispersed Poisson [1] or negative binomial models [2], [3].

In order to study the relationships between these large numbers of genes, several authors have worked on co-expression networks and used methods based on Pearson correlation [4] or canonical correlation [5], [6], but no specific models have been designed for RNA-seq data. A further question is how these genes interact with each other. Inference of gene networks from transcriptomic data is indeed a key aspect of systems biology that may help unravel and better understand the underlying biological regulatory mechanisms. Various models have been proposed for network inference from microarray data, mainly based on Gaussian graphical models [7], [8]. Until now, very few authors have addressed the question of network inference from RNA-seq data. Some authors simply use methods based on a Gaussian assumption for RNA-seq data [9]. We propose in this paper to compare various approaches to tackle this issue.

The simplest idea is to perform an appropriate transformation of the data, using for example a Box-Cox transformation [10] and apply methods that rely on an assumption of normality. Another possibility is to use models specifically designed for count data with large variability. Allen and Liu [11] recently proposed a Poisson log-linear graphical model adapted to count data. This model requires a power transformation of the data [12] when the inter-sample variance is greater than the sample mean. We propose in this paper a hierarchical log-normal Poisson model with a Lasso penalty, which has the advantage of directly modelling inter-sample variability and can therefore be readily applied to the raw data. Performance of these different methods for gene network inference are compared on data simulated under a multivariate Poisson distribution [13] with various amounts of additional inter-sample variability, as well as on publicly available microRNA-seq data collected on breast invasive carcinoma (BRCA) tumors, downloaded from The Cancer Genome Atlas (TCGA) Data Portal.

## Materials and Methods

We first define the notation that will be used throughout this paper. Let *Y_ij_* be the random variable corresponding to the gene expression measure for the sample *i* (*i* = 1, …, *n*) for the gene *j* (*j* = 1, …, *p*), with *y_ij_* being the corresponding observed value of *Y_ij_*. Note that *i* always indexes samples and *j* always indexes genes with *n* the number of samples and *p* the number of genes. A network represents gene interactions. The nodes are random variables modelling the gene expression levels and the edges indicate the dependencies between those variables. In this section we provide a short description of the models that will be compared for gene network inference from RNA-seq data.

### Gaussian graphical model

The underlying assumption of this model is that the data are normally distributed. In the case of untransformed RNA-seq data, this assumption is not valid since data counts cannot take negative values. We investigated a variety of Box-Cox transformations to lead to approximately normal data [10], where the *δ* value was chosen to maximize the log-likelihood of the transformed data: 
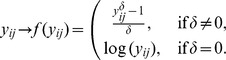



Since gene expression data may contain zero counts, we usually use (*y*+1) instead of *y* in the Box-Cox formula above. Let 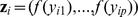 be the transformed vector of expression values for *p* genes for the *i* th biological sample (*i* = 1, …, *n*). We assume that 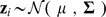. The edges of the inferred network correspond to non-zero partial correlations, i.e. the non-zero elements of matrix 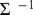 [7], [14].

Let ***S*** be the empirical covariance matrix. The log-likelihood of the model is: 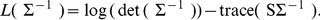
(1)


A common assumption in the context of gene networks is that the matrix 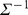 is sparse. We add an 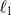 penalty to the log-likelihood (1) so that some coefficients in the estimated 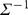 matrix are precisely equal to 0: 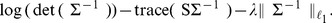
(2)


Network inference using a Gaussian graphical model has been extensively studied and used over the past years. Many methods exist to compute the penalized maximum likelihood estimate of the **Σ** matrix above. We use the method implemented in the glasso R package [7] which makes use of a coordinate descent algorithm.

The choice of the regularization parameter *λ* has also been extensively studied [15]. We choose to perform model selection by maximizing the Bayesian Information Criterion (BIC) [16] defined below, where *ν* represents the number of free parameters in the model: 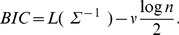
(3)


Note that a single parameter *λ* is chosen for the entire network.

### Log-linear Poisson graphical model

A log-linear Poisson graphical model specifically designed for network inference from count data has been recently proposed [11]. This model is based on a Poisson distribution which assumes the mean and variance to be equal. Therefore, the model does not account for the high dispersion of the data, also called over-dispersion with respect to the Poisson distribution, when the sample variance is higher than the sample mean. To apply it to RNA-seq data, the authors propose to use a power transformation of the data 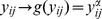, with 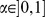 implemented in the R package PoiClaClu [12]. The coefficient *α* is chosen to maximize an adequacy criterion between the transformed data 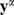 and a Poisson distribution.

Let 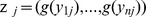 be the transformed vector of expression values for gene *j* in the *n* biological samples. It is assumed that the conditional distribution of *Z_ij_* given all the other genes 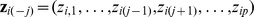 is a Poisson distribution 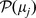, with 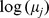 modelled as a linear regression on all the other genes: 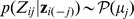



with 
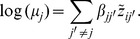



The notation 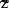 corresponds to a standardization of the log-transformed data. This standardization is a necessity since we model the mean of the gene *j* and not the random variable itself. An edge is present in the inferred graph if one or both parameters 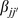 and 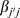 are different from zero. The log-likelihood for gene *j* can be written in this case as: 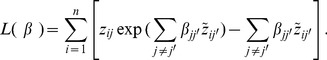
(4)


Similar to the previous model, we assume that the vector 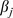 is sparse. We add an 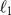 penalty to the log-likelihood (4) so that some coefficients in the estimated 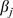 vector are set to 0. Estimation of parameters 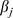 can be obtained by a coordinate gradient algorithm as implemented in the R package glmnet [17]. We propose to perform the model selection with the Stability Approach to Regularization Selection creterion (StARS), as suggested by [11]. This stability-based method selects the network with the smallest amout of regularization that simultaneously makes the network sparse and replicable under random sampling. Note that we select only one regularization parameter for all the regressions in the network problem.

### Hierarchical log-normal Poisson graphical model

We note that the Poisson model presented above requires a transformation of the data to account for the high dispersion. Here we propose to deal with it directly with a hierarchical log-normal Poisson model. The count expression of gene *j* for sample 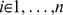 is modeled as: 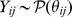 with 
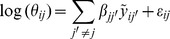


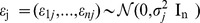



As before, the notation 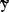 corresponds to a standardization of the log-transformed data. Here, the vector 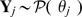 and 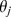 is itself a random variable: 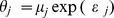 with 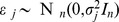 and 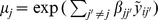. Note that the variance of the random variable 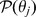 is larger than its mean if 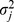 is positive. As previously, an edge is present in the graph between genes *j* and 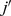 if one or both parameters 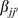 and 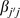 are different from zero.

In this model, the likelihood for gene *j* can be written as: 
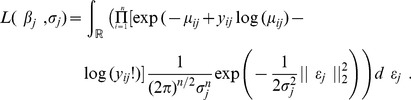
(5)


Similar to the previous model, we assume that the vector 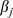 is sparse. We add an 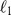 penalty to a function of the log-likelihood (5) so that some coefficients in the estimated 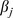 vector are set to 0: 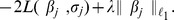



Estimation of parameters 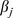 and 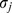 was done using the R function glmmixedlasso [18], based on a Laplace approximation of the penalized likelihood and a coordinate descent algorithm.

An important aspect of this method is the choice of the regularization parameter *λ*. To choose a common *λ* parameter for all the gene-by-gene regressions, we propose to use a two stage approach for this parameter. First, for each gene *j*, a 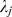 parameter is chosen by maximizing the BIC criterion defined as 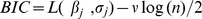, where 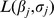 is the unpenalized log-likelihood and *ν* is the number of free parameters in the model. Then the mean of the 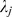 parameters is taken as the regularization parameter and used for all the regressions: 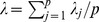. Since BIC is an asymptotic criterion, taking the average of the regularization parameters over all the regressions helps to improve network inference performance.

## Results

### Simulation study

#### Multivariate Poisson data simulation

In order to simulate multivariate Poisson data, we use a method described by Karlis [13]. As an illustration, for a two dimensional multivariate Poisson distribution, we simulate three independent Poisson variables 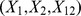 and sum them up (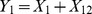 and 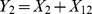) so that the resulting variables are not independent: 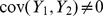 if 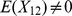. In the general case, a sample **y** of dimension 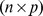 where *p* is the number of nodes in the network, *n* the number of samples is obtained by summing samples from 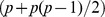 independent Poisson random variables. The adjacency matrix 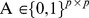 encodes the underlying graph structure: 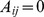 means that the expression level of genes 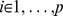 and 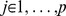 are conditionally independent given the other gene expression levels. In order to sum the 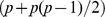 terms accordingly, we fix the matrix ***B*** of dimension 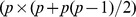: 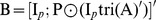 where ***P*** is a permutation matrix of dimension (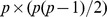) of vector (1,1,0,…,0), 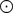 denotes the matrix multiplication element by element and 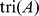 is the vector of dimension 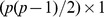 containing the elements of the upper triangular adjacency matrix. The matrix product 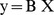 gives a count data table of size 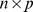: *n* samples from a *p*-dimensional Poisson random variable whose underlying dependency structure is encoded in the known ***A*** matrix.

RNA-seq data are known to be overdispersed relative to a Poisson distribution with the sample variance of a gene expression vector larger than the sample mean. In our simulation study, we also consider the possibility of inflating the variance of the independent Poisson random variables used in the ***X*** matrix of the formula above by simulating independent variables according to a log-normal Poisson model. For gene *j* and sample *i*, we sample 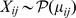 with 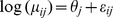, 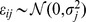. We use this log-normal Poisson distribution only for the first *p* columns of the matrix, the other columns being sampled from a simple Poisson distribution.

#### Simulation settings

The three methods were compared on two sets of simulations: multivariate Poisson data and overdispersed multivariate Poisson. For each type of data, we simulated 50 different adjacency matrices ***A*** with a scale-free structure. This implies that degrees of the edges are assumed to follow a power law distribution, i.e. few nodes in the network are well connected and most of the nodes have only one or two neighbours. The number of nodes *p* was set to 50. With a scale-free structure, the maximum degree of a node is 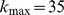 and the average degree is less than 2. To avoid the ultra-high dimensional setting, defined as 
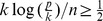
 for Gaussian linear regression [19], we set the number of biological samples to *n* = 100. For each of the 50 different adjacency matrices, 1225 samples of size *n* were simulated from Poisson random variables (adding extra inter-sample variance or not) and summed up as explained above to obtain the final data set of size 100×50. We chose to use Poisson distributions of mean *μ* = 100 to build the ***X*** data matrix, resulting in data counts ranging from around 100 to 2500. In the case of Poisson data with inflated variance, the parameter 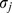 was set to 0.25, which is slightly smaller than the amount of dispersion observed in the real data presented below.

To evaluate the different methods, we tried to infer the adjacency matrix ***A*** from the simulated dataset 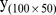 and compared the inferred matrix 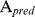 with the real adjacency matrix ***A*** used to simulate the data. For each type of data (with and without extra inter-sample variance) and for each network inference method (Gaussian, log-linear Poisson, and the proposed hierarchical log-normal Poisson graphical models), Receiver Operating Characteristic (ROC) curves were constructed by varying values of the regularization parameter from an empty network (sensitivity equal to 0) to a full network (specificity equal to 0). The sensitivity and specificity values were also compared for the different methods using the chosen regularization parameter (with the BIC criterion for the Gaussian graphical model, StARS criterion for the log-linear Poisson graphical model and the mean-BIC criterion presented above for the hierarchical log-normal Poisson model). Note that in the case of the Poisson graphical model, a power transformation is applied only in the simulation setting inducing inflated variance.

#### Results

**Figure 1:**
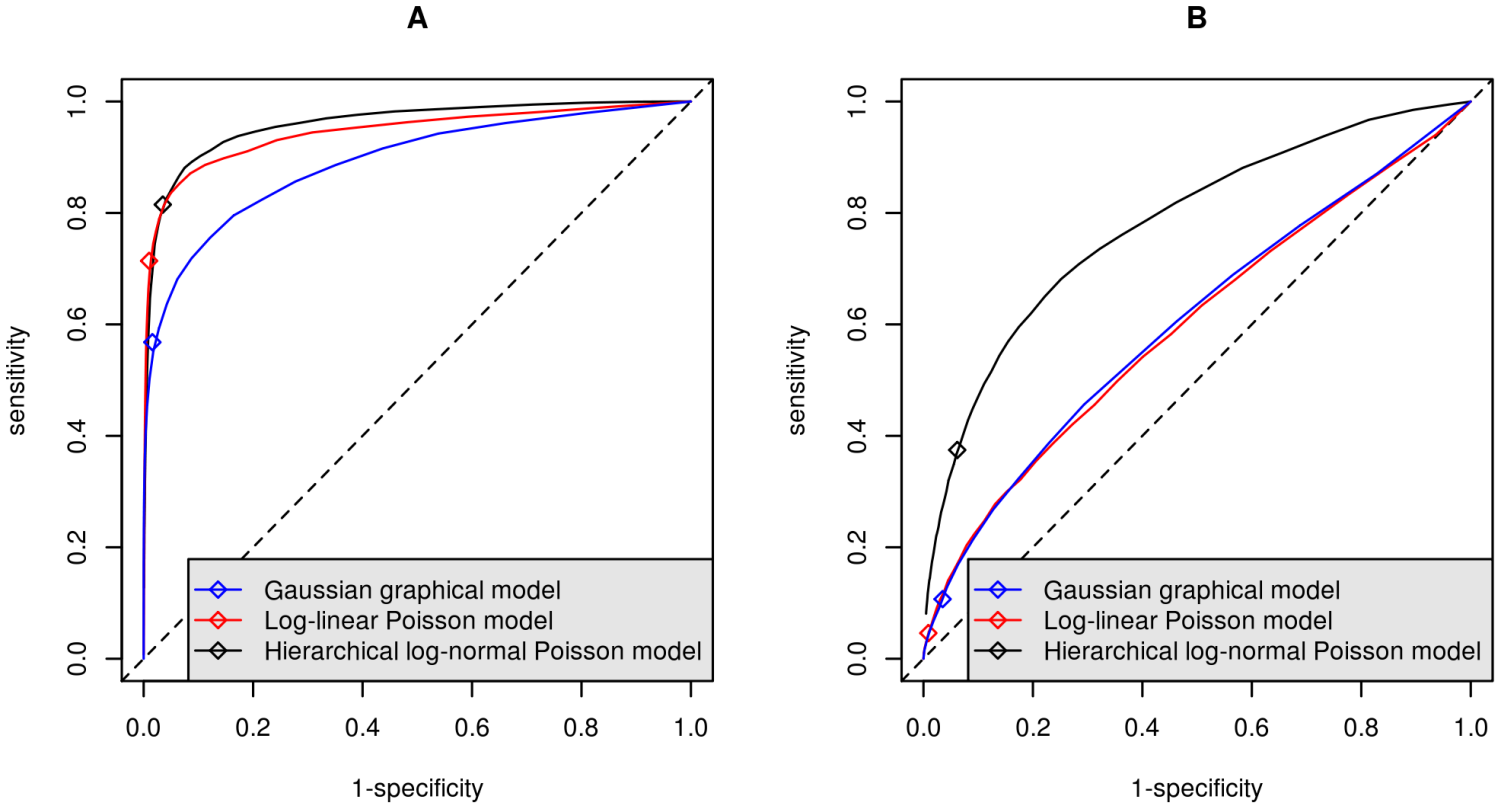
ROC curves, averaged over 50 simulated data sets on scale-free graphs. Results are presented for the Gaussian graphical model on log-transformed data (blue), the log-linear Poisson graphical model on power-transformed data (red) and the hierarchical log-normal Poisson model on raw data (black) on multivariate Poisson data (A) and multivariate Poisson data with inflated variance (B). The dotted black lines represent the diagonals.

ROC curves, averaged over the 50 simulated datasets, are presented in Figures 1 for the two simulation settings (multivariate Poisson data with or without inflated variance). It can be noticed that in the first setting, with no over-dispersion, the log-linear Poisson model outperforms the Gaussian graphical model applied to transformed data. This result was already observed [11]. As expected, in this case the performance of the log-linear Poisson model and the proposed hierarchical model are very similar. When adding extra variability to the data, we are compelled to use a power-transformation of the data to apply the log-linear Poisson model [11], since the data no longer respect the Poisson assumption of equal mean and variance. The performance of the log-linear Poisson model in this case is considerably deteriorated, and is now comparable to the poor performance of the Gaussian graphical model on log-transformed data. The proposed hierarchical log-normal Poisson model therefore outperforms the two other methods in this case, keeping in mind that the data were simulated under a closely related model that was deemed to be a reasonable choice to approximate the dynamics of RNA-seq data. It has to be pointed out that for the over-dispersed data, performances of the three methods are considerably worse compared to the simple case of multivariate Poisson data due to the presence of additional variability.

Sensitivity and specificity obtained by each method for the chosen regularization parameters are represented in diamond-shape squares on the ROC curves (Figures 1) and are summarized in Table 1. The regularization parameter chosen with the mean-BIC criterion for the proposed hierarchical log-normal Poisson model offers a higher sensitivity than the Poisson or Gaussian graphical models, even when no over-dispersion was simulated (0.84 compared to 0.71 and 0.57, respectively), while keeping a high specificity (0.97 compared to 0.99 and 0.98, respectively). The number of correctly detected edges is therefore larger for the proposed model compared to the other two methods, even in the case of multivariate Poisson data with no over-dispersion. When adding extra inter-sample variability, the differences between the three methods are even larger, even if the performances deteriorate for all methods (sensitivity equal to 0.4 for the proposed model compared to 0.1 for the Gaussian graphical model and 0.05 for the Poisson graphical model). These very low sensitivity values can partly be explained by the fact that scale-free structures were considered for the simulated graphs, therefore generating only a small number of edges compared to a random graph structure that are difficult to correctly detect. This also explains, on the other hand, the high specificity values. In fact, as the models infer very few edges for low numbers of biological replicates, they have less chance to detect incorrect edges. Both the ROC curves and the sensitivity/specificity for the chosen regularization parameter therefore show much better performances for the proposed hierarchical model than the Gaussian graphical model on log-transformed data or the Poisson graphical model on power-transformed data, especially in the case of overdispersed multivariate Poisson data.

**Table 1:** Average sensitivity and specificity (standard deviation in parentheses) for the selected network across 50 simulated networks with scale-free structure.

		GGM	Log-linear Poisson	Hierarchical model
Multivariate Poisson Data	Sens.	0.568 (0.069)	0.714 (0.036)	0.838 (0.050)
	Spec.	0.984 (0.003)	0.990 (0.003)	0.967 (0.006)
Over-dispersed Poisson Data	Sens.	0.107 (0.045)	0.046 (0.033)	0.383 (0.064)
	Spec.	0.965 (0.003)	0.991 (0.004)	0.982 (0.027)

Results are averaged over 50 datasets for multivariate Poisson data and overdispersed multivariate Poisson data. GGM: Gaussian graphical model on transformed data (log(y+1)), Log-linear Poisson: log-linear Poisson graphical model proposed by [11] on power transformed data (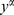), Hierarchical model: proposed model as detailed in the Methods section and applied on the raw data.

**Figure 2:**
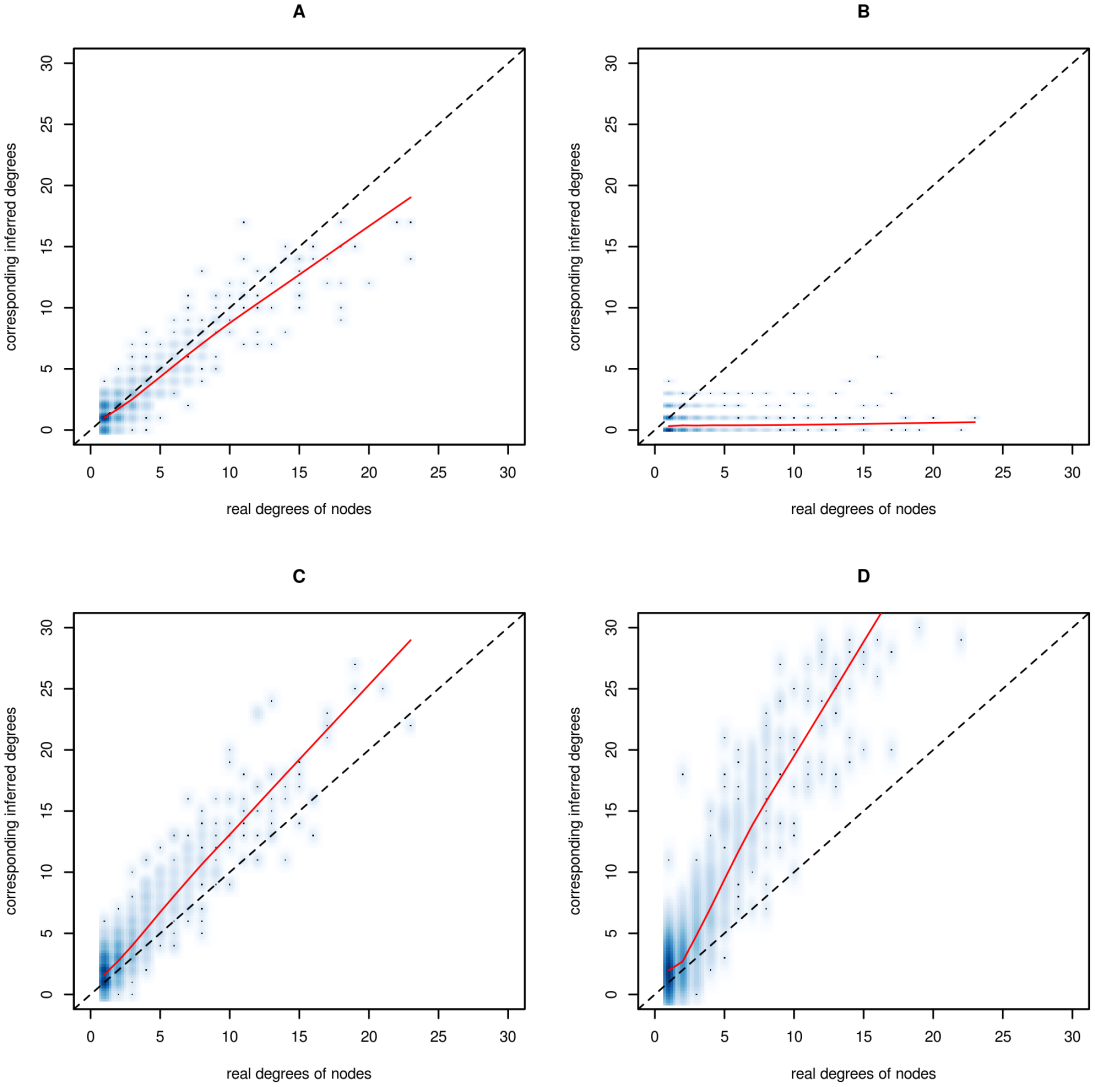
Relationship between the degree of the nodes in the estimated network and in the simulated network on scale-free graphs. Results are presented for the log-linear Poisson graphical model without over-dispersion (A) and with over-dispersion (B), for the proposed hierarchical log-normal Poisson graphical model without over-dispersion (C) and with over-dispersion (D). Black dotted lines represent the diagonal, and red lines represent loess curves.

Figures 2 represents the relationships between the degree of the nodes in the estimated network and in the simulated structure for both the Poisson graphical model and the proposed hierarchical model. It can be observed that, as expected, in the case of no over-dispersion, both methods perform quite similarly, as already seen in the ROC curves above. In the case of over-dispersion, however, even if the sensitivity was quite poor for all methods (Table 1), the structure of the graph was much better preserved with the proposed model than with the Poisson graphical model on power transformed data.

**Figure 3:**
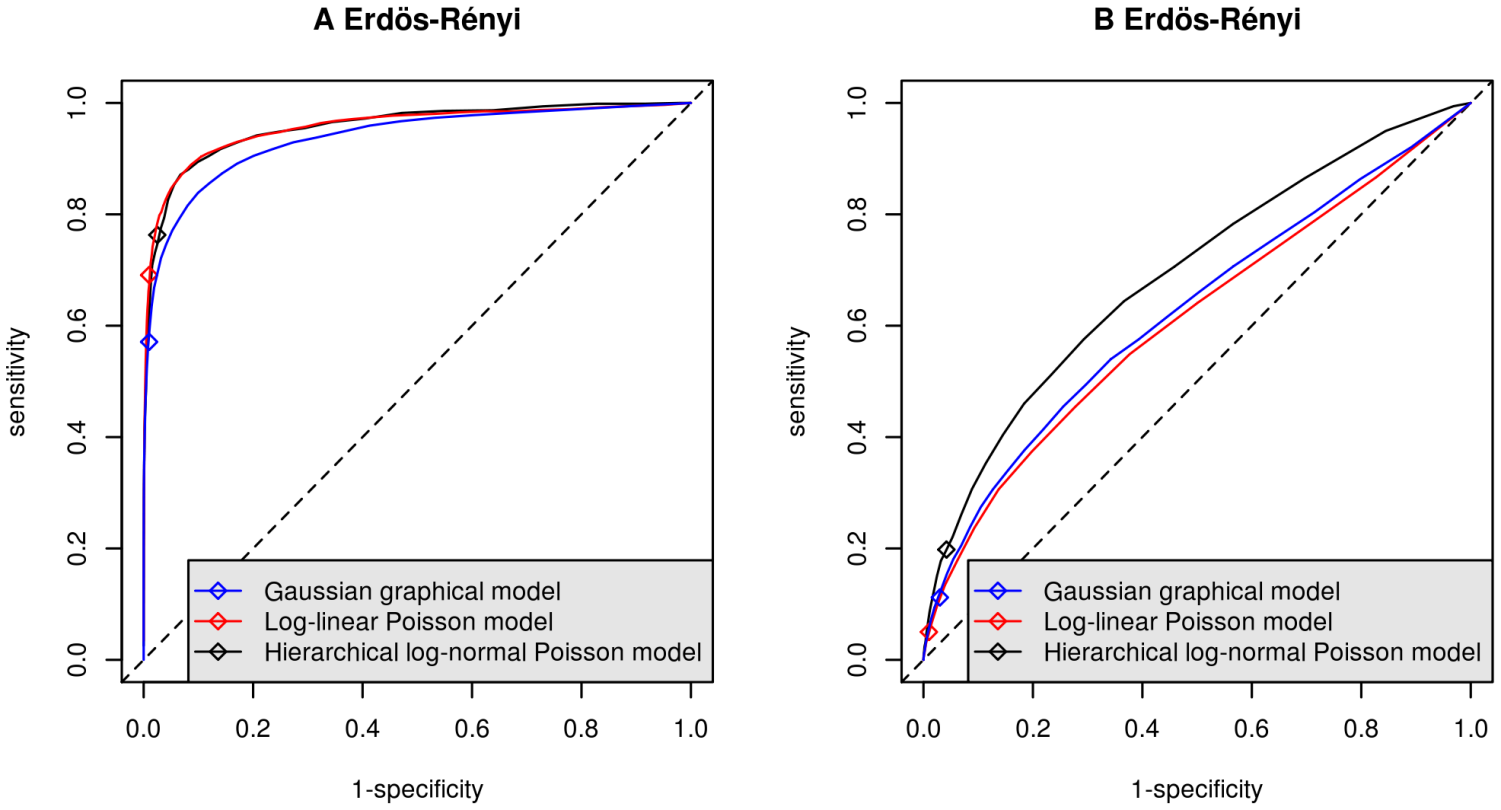
ROC curves, averaged over 30 simulated data sets on Erdös-Rényi graphs. Results are presented for the Gaussian graphical model on log-transformed data (blue), the log-linear Poisson graphical model on power-transformed data (red) and the hierarchical log-normal Poisson model on raw data (black) on multivariate Poisson data (A Erdös-Rényi) and multivariate Poisson data with inflated variance (B Erdös-Rényi). The dotted black lines represent the diagonals.

To ensure that these results do not depend on the scale-free structure of the graphs, we have drawn ROC curves and performed similar model selection on data simulated with an Erdös-Rényi structure [20] (Figures 3 and Table 2). For Erdös-Rényi graphs, each pair of nodes are connected with the same probability, independently of the other pairs of nodes. Although the differences among the three methods are less pronounced for Erdös-Rényi structures than for scale-free structures as previously observed [11], the same general conclusions hold.

**Table 2:** Average sensitivity and specificity (standard deviation in parentheses) for the selected network across 30 simulated networks with Erdös-Rényi structure.

		GGM	Log-linear Poisson	Hierarchical model
Multivariate Poisson Data	Sens.	0.571 (0.059)	0.691 (0.061)	0.763 (0.093)
	Spec.	0.992 (0.003)	0.990 (0.003)	0.975 (0.005)
Over-dispersed Poisson Data	Sens.	0.112 (0.065)	0.050 (0.041)	0.198 (0.060)
	Spec.	0.971 (0.003)	0.990 (0.003)	0.958 (0.009)

Results are averaged over 30 datasets for multivariate Poisson data and overdispersed multivariate Poisson data. GGM: Gaussian graphical model on transformed data (log(y+1)), Log-linear Poisson: log-linear Poisson graphical model proposed by [11] on power transformed data (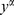), Hierarchical model: proposed model as detailed in the Methods section and applied on the raw data.

### Real data analysis

#### Data description

The three methods were applied to a publicly available microRNA-seq data set available at The Cancer Genome Atlas (TCGA) Data Portal (http://cancergenome.nih.gov/). We selected 100 samples from breast invasive carcinoma (BRCA) tumors. To avoid being in an ultra high-dimensionality setting [19], we reduced the number of microRNAs used for network inference to 50 (among 863). To do so, we first removed all microRNAs that had at least one null count. Among the remaining 207, we selected the microRNAs with the largest inter-sample variance (as suggested by [11]). These microRNAs are the most likely to be linked to breast cancer development since they are selected among the most highly variable microRNAs. Note that we did not perfom any normalization for differences in library sizes on this data set, as contrary to differential analyses [2], [21], differences in library sizes have no impact on the network inference results since we do not compare two different biological samples, but relate the expression of genes within each biological sample. Since each miRNA has an equal number of nucleotides, there is no need for a gene length correction either.

#### Modelling the data

**Figure 4:**
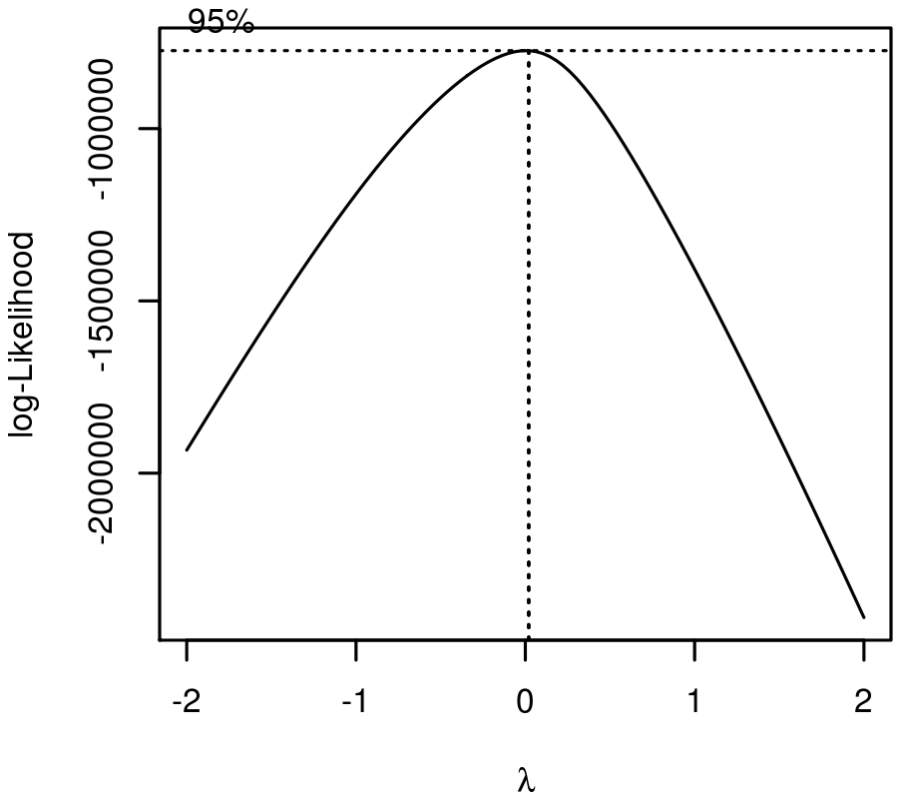
Optimal parameter for the Box-Cox transformation of data. Curve obtained with the R package MASS.

Shapiro-Wilk tests on miRNA expression vectors showed that the data, even for highly expressed miRNAs, could not be directly modelled as a normal distribution [22]. We therefore used a Box-Cox transformation [10] prior to applying a Gaussian graphical model to these data. The optimal Box-Cox parameter to make the data as normally distributed as possible was found to be close to zero, which corresponds to a log-transformation of the data (Figure 4).

**Figure 5:**
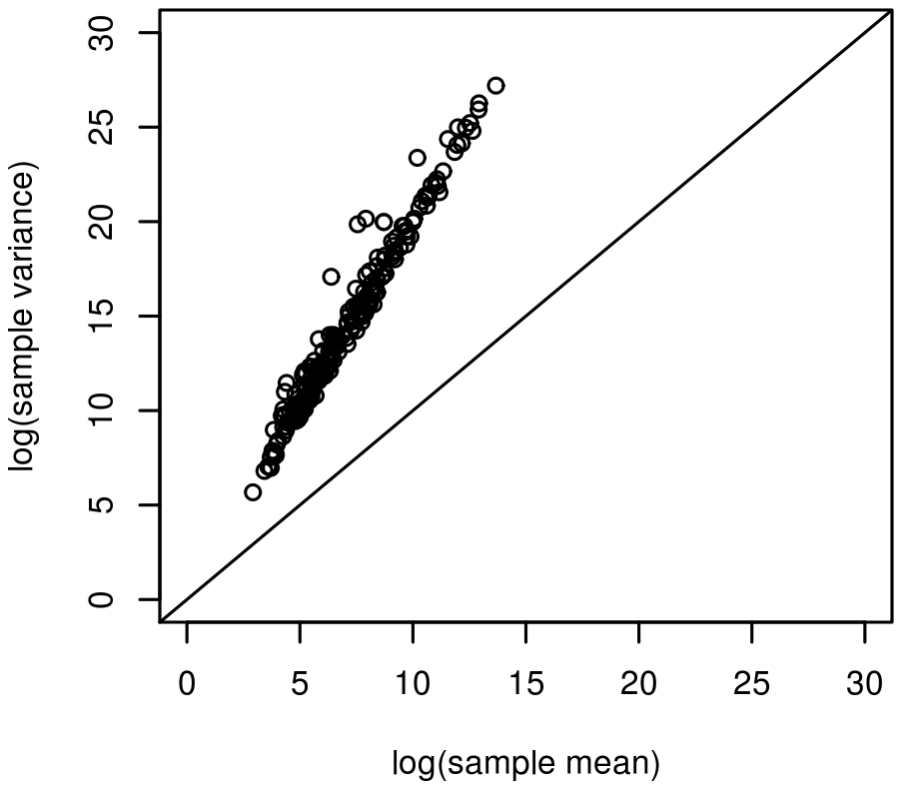
Sample mean-variance relationship for the 207 microRNAs.

For these data, the Poisson assumption is not verified either, as shown in Figure 5, since the sample variance is considerably larger than the sample mean for all miRNAs. As suggested in [11], we therefore applied the power-transformation implemented in the PoiClaClu package prior to applying the log-linear Poisson graphical model.

**Figure 6:**
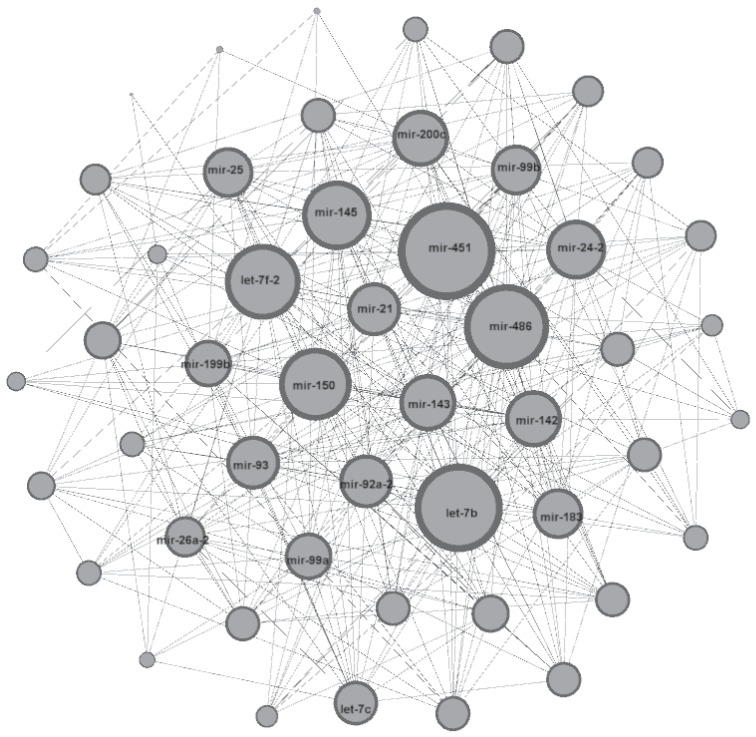
Network inferred with the hierarchical model. The representation was obtained using the software Gephi [25]. The size of nodes represents the number of edges associated with the corresponding gene in the network.

The Gaussian graphical model with the BIC criterion detected 48 edges, the log-linear Poisson graphical model with the StARS criterion [11] detected 74 edges, and the proposed hierarchical log-normal Poisson graphical model detected 369 edges among the 50 miRNAs considered here. As shown in Figure 5, these data exhibit significant over-dispersion with respect to the Poisson assumption. We are therefore close to the second simulation setting presented above. In this case, the sensitivity of the proposed hierarchical model is expected to be much higher than for the other two methods, which explains the much larger number of detected edges. Figure 6 presents the network inferred by the hierarchical model. Table 3 presents the biological functions of the most highly connected nodes found with the proposed hierarchical model. It can be noticed that a large majority of these miRNAs are already known to be related to breast cancer. Further biological validation would be interesting for the remaining ones that could be new potential therapeutic targets.

**Table 3:** Ten most highly connected genes in the network inferred by the proposed hierarchical model.

miRNA	reference
hsa-mir-451	BC [26]
hsa-let-7b	BC [27]
hsa-mir-486	BC [28]
hsa-let-7f-2	cancer [27]
hsa-mir-150	no reference
hsa-mir-145	BC [29]
hsa-mir-24-2	BC [30]
hsa-mir-200c	BC [31], [27]
hsa-mir-143	BC [32]
hsa-mir-142	no reference

BC corresponds to miRNAs known to be linked to Breast Cancer, with the corresponding references.

## Discussion

Network inference from RNA-seq data is an important methodological challenge. This work is a pioneer study to provide some guidelines on the best methods to achieve this goal. There are two main approaches. The first and simplest idea is to perform a transformation of the data and apply previously proposed methods for microarray studies based on Gaussian graphical models, for example using a Box-Cox transformation. Another possibility is to apply methods specifically developed for the analysis of count data using Poisson graphical models, either with a power transformation of the data or by accounting for over-dispersion directly in the model using for example a hierarchical log-normal Poisson graphical model as proposed here. We found in both simulation study and real data application that the power transformation did not work well to correct for over-dispersion. It has to be noted that the same *α* parameter was used here for all the genes. It might be possible to improve the performance of this method if a different coefficient was estimated for each gene. This is, however, not possible with the method proposed by [23], which finds the optimal value by maximizing the adequacy criterion for a group of genes. In this work the best suited methodology for network inference from RNA-seq data currently appears to be the proposed hierarchical Poisson log-normal model, which seems to be able to appropriately deal with highly dispersed count data. However, the implementation of this approach based on the R package glmmixedlasso [18] is quite slow for a large number of biological samples and more research is needed to optimize this function.

It has to be pointed out that in high-dimensional settings (number of genes much larger than the number of biological samples), all methods were unsurprisingly found to perform very poorly, despite the 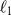 regularization. As for microarray studies, the limited number of biological replicates available in RNA-seq experiments considerably restrains the number of genes that can be included in the network. Future research is needed to tackle this issue. A first possibility may be to try to reduce the number of parameters to be estimated. In fact, in a first step we aim at finding the regulatory relationships between genes without necessarily estimating their strength precisely. Therefore, in the regression models presented above, instead of trying to estimate one parameter for each gene we could infer parameters for groups of genes. Alternatively, to face the problem of small numbers of biological replicates, instead of inferring regulatory networks within each experimental condition, it would be interesting to use joint graphical model approaches [24] to jointly infer a network in multiple conditions, thus highlighting the common or differing patterns across conditions.
